# Cancer‐Control Outcomes of Japanese Vs. German Metastatic Hormone‐Sensitive Prostate Cancer Patients: A Real‐World Cross‐Country Comparison of Patients Undergoing Combination Therapies

**DOI:** 10.1111/iju.70633

**Published:** 2026-09-09

**Authors:** Mike Wenzel, Keiichiro Miyajima, Maximilian Filzmayer, Axel S. Merseburger, Carolin Siech, Benedikt Lauer, Felix K.‐H. Chun, Shin Koike, Yoichiro Tohi, Takafumi Yanagisawa, Kojiro Tashiro, Takahiro Kimura, Fumihiko Urabe, Philipp Mandel

**Affiliations:** ^1^ Department of Urology Goethe University Frankfurt, University Hospital Frankfurt Germany; ^2^ Department of Urology The Jikei University School of Medicine Tokyo Japan; ^3^ Department of Urology University Hospital Schleswig‐Holstein, Campus Lübeck Lübeck Germany; ^4^ Martini‐Klinik Prostate Cancer Center and Department of Urology University Hospital Hamburg‐Eppendorf Hamburg Germany; ^5^ Department of Urology Itabashi Chuo Medical Center Itabashi Japan; ^6^ Department of Urology, Faculty of Medicine Kagawa University Takamatsu Japan

**Keywords:** Germany, Japan, metastatic prostate cancer, mHSPC, survival

## Abstract

**Objectives:**

Differences in tumor biology, ethnicities, and healthcare structures across populations may contribute to variability in oncologic outcomes. This study compared baseline characteristics and oncologic outcomes of Japanese and German metastatic hormone‐sensitive prostate cancer (mHSPC) patients.

**Methods:**

A pooled analysis of 1 215 mHSPC patients from Japan (JIKEI‐YAYOI database, *n* = 639) and Germany (FRAMCAP database, *n* = 576) undergoing combination therapies between 2015 and 2025 was performed. Cox regression models were used to evaluate time to castration‐resistant prostate cancer (ttCRPC) and overall survival (OS). Propensity score matched analyses (PSM) validation was performed.

**Results:**

Japanese patients were older at diagnosis (median 73 vs. 70 years) and had higher PSA at mHSPC diagnosis (260 vs. 43 ng/mL). Moreover, Japanese patients had more frequently synchronous mHSPC (95% vs. 75%), high‐volume disease (78% vs. 54%), and visceral metastases (16% vs. 8%). ADT plus androgen receptor pathway inhibitors (ARPI) was used more frequently in Japan (76% vs. 71%), as was the combination of ADT + ARPI + docetaxel (17% vs. 11%). Conversely, ADT + docetaxel was less commonly used in Japan (7% vs. 19%). Median ttCRPC was 39 vs. 23 months, and median OS was 80 vs. 51 months (Japan vs. Germany). After adjustment for relevant clinical covariates, Japanese cohort affiliation remained an independent predictor of prolonged ttCRPC (HR 0.53, *p* < 0.001) and improved OS (HR 0.54, *p* < 0.001). PSM analyses confirmed similar findings.

**Conclusions:**

Despite presenting with more adverse baseline characteristics, Japanese mHSPC patients demonstrated longer ttCRPC and improved OS compared with patients in Germany. These differences may reflect not only potential biological variations but also differences in treatment strategies and healthcare structures.

**Trial Registration:**

Not applicable

**Abbreviations**
ADT: androgen deprivation therapy
ARPI: androgen receptor pathway inhibitor
CI: confidence interval
CT: computed tomography
EAU: European Association of Urology
ECOG: Eastern Cooperative Oncology Group Performance Status
FRAMCAP: FRAnkfurt Metastatic CAncer database of the Prostate
HR: hazard ratio
IQR: interquartile range
mCRPC: metastatic castration‐resistant prostate cancer
mHSPC: metastatic hormone‐sensitive prostate cancer
OS: overall survival
PARP: poly(ADP‐ribose) polymerase
PET: positron emission tomography
PSA: prostate‐specific antigen
PSMA: prostate‐specific membrane antigen
RECIST: Response Evaluation Criteria in Solid Tumors
RLT: radio‐ligand therapy
ttCRPC: time to metastatic castration‐resistant prostate cancer

## 1 Introduction

Metastatic hormone‐sensitive prostate cancer (mHSPC) represents a biologically heterogeneous disease with variability in clinical presentation, metastatic patterns, and oncologic outcomes [1, 2]. Over the past decade, treatment strategies for mHSPC have evolved substantially with the introduction of systemic therapy intensification beyond androgen deprivation therapy (ADT) [3, 4, 5, 6, 7, 8, 9, 10]. Randomized trials have demonstrated significant survival benefits with the addition of androgen receptor pathway inhibitors (ARPI) or taxane‐based chemotherapy, with subgroup analyses suggesting comparable treatment effects in Asian populations [6, 7, 8, 9, 10, 11, 12, 13, 14]. Consequently, contemporary international guidelines, including the European, German, Asian‐Pacific, and Japanese guidelines for prostate cancer, recommend treatment intensification with ARPI‐based doublet or taxane‐containing triplet therapy in eligible patients with mHSPC [15, 16, 17].

Despite broadly similar therapeutic principles across these guidelines, previous studies have suggested that tumor biology, metastatic burden, and patterns of disease dissemination may vary between Western and Asian populations [18, 19]. Moreover, variations in clinical practice patterns, healthcare infrastructure, and guideline implementation may further contribute to differences in oncologic outcomes across regions.

Comparative international real‐world analyses may therefore provide important insights into how population characteristics and treatment strategies differ between patients in Japan and Germany and to what extent these potential differences translate into clinical outcomes. In this context, we conducted a pooled analysis of two large real‐world mHSPC databases from Japan and Germany.

## 2 Methods

### 2.1 Study Population

After obtaining approval from the local ethics committees and in accordance with the principles of the Declaration of Helsinki, we retrospectively identified patients with mHSPC from the FRAnkfurt Metastatic CAncer database of the Prostate (FRAMCAP, reference no. SUG‐5‐2024, tertiary care hospital, single center database) and the JIKEI‐YAYOI database (reference no. 37–562‐13 219), which includes data from four academic centers within the Jikei University system and 12 affiliated community‐based hospitals. Identical inclusion criteria were applied to the German and the Japanese cohorts. Eligible patients were required to have synchronous or metachronous mHSPC with histologically confirmed prostate cancer and radiologically confirmed metastatic disease, and initiation of systemic treatment for mHSPC. Patients were included irrespective of metastatic site, including bone, lymph node, or visceral metastases. Only patients were included with combination mHSPC treatment of ADT plus docetaxel or ARPI or both starting from 2015 until 2025. Staging was performed using either conventional imaging with computed tomography (CT) and bone scintigraphy or prostate‐specific membrane antigen (PSMA) positron emission tomography (PET) with CT. Baseline clinical characteristics, metastatic patterns, and treatment information were extracted from both databases. After applying the predefined inclusion criteria, a total of 1 215 patients with mHSPC were included in the final study population, comprising 639 patients from the JIKEI‐YAYOI database (Japan) and 576 patients from the FRAMCAP database (Germany).

### 2.2 Study Endpoints

The main study endpoints were time to castration‐resistant prostate cancer (ttCRPC) and overall survival (OS). ttCRPC was defined as time from initiation of therapy for mHSPC to the development of metastatic castration‐resistant prostate cancer (mCRPC) or initiation of subsequent systemic treatment or death. mCRPC was defined according to EAU (European Association of Urology) guidelines [15]: Three consecutive rises in prostate‐specific antigen (PSA) values during mHSPC treatment, with a PSA level above 2 ng/mL and a 50% rise above the nadir. OS referred to the time from treatment initiation to death due to any cause.

### 2.3 Statistical Analyses

Descriptive statistics encompassed frequencies and proportions for categorical variables. Medians and interquartile ranges (IQR) were reported for all continuously coded variables.

To assess group differences, the Wilcoxon rank‐sum test was used for continuous or ordinal variables without normal distribution. Pearson's Chi‐squared test was applied to evaluate differences in proportions for categorical variables. For all survival analyses, Kaplan–Meier curve‐derived estimates with log‐rank tests, as well as univariable and multivariable Cox proportional hazards models, were applied. Multivariable regression models were fitted adjusting for year of mHSPC treatment initiation, age at mHSPC diagnosis, Eastern Cooperative Oncology Group (ECOG) performance status, first‐line systemic treatment (ARPI, docetaxel, or ARPI and docetaxel), PSA serum level at diagnosis, local treatment of the primary tumor, disease volume according to CHAARTED criteria (high vs. low), and synchronous vs. metachronous mHSPC disease. For OS, additional adjustment for the number of systemic therapy lines for metastatic prostate cancer was performed. Variables were selected a priori based on established clinical relevance and low collinearity, while additional correlated variables were excluded to avoid model overfitting (e.g., Gleason/ISUP score). The proportional hazards assumption was assessed using Schoenfeld residuals.

Based on the same set of covariates, propensity score matching was performed as a sensitivity analysis using 1:1 nearest‐neighbor matching with a caliper of 0.2. Time‐to‐event analyses in the matched cohort were repeated using Cox regression models with robust standard errors accounting for matched subclasses. Both unadjusted and additionally covariate‐adjusted models (doubly robust approach) were evaluated.

All tests were two‐sided with a level of significance set at *p* < 0.05. R software environment for statistical computing and graphics (version 3.4.3) was used for all analyses.

## 3 Results

### 3.1 Study Population and Baseline Characteristics

Overall, 1 215 mHSPC patients could be included in the current analyses, with a median follow‐up of 23 months in both countries (Table 1). Median age at mHSPC stage was 74 years (IQR: 69–79) among patients in Japan and 70 years (IQR: 64–76, *p* < 0.001) among patients in Germany. Median PSA at mHSPC was higher in the Japanese cohort (260 ng/mL, IQR: 74–890 ng/mL) compared to the German cohort (43 ng/mL, IQR: 10–240 ng/mL, *p* < 0.001). Patients in Japan presented with a higher prevalence of high volume mHSPC according to CHAARTED criteria (77.6% vs. 54.1%, *p* < 0.001) and LATITUDE high risk often (77.4% vs. 63.4%, *p* < 0.001), visceral metastases (16.3% vs. 7.6%, *p* < 0.001), and synchronous mHSPC (95.1% vs. 74.7%). No differences were observed in the distribution of ECOG status.

**TABLE 1:** Descriptive characteristics of 1 215 metastatic hormone‐sensitive prostate cancer patients stratified according to treatment in Japan vs. Germany.

Characteristic	*N*	Overall *N* = 1 215^a^	Japan *N* = 639 (52.6%)^a^	Germany *N* = 576 (47.4%)^a^	*p* ^b^
Year of mHSPC treatment initiation	1 215	2022 (2020, 2023)	2022 (2020, 2023)	2021 (2019, 2023)	< 0.001
Age at mHPSC diagnosis (years)	1 215	72 (66, 78)	74 (69, 79)	70 (64, 76)	< 0.001
ECOG status	1 041				0.3
0		722 (69.4%)	445 (69.9%)	277 (68.6%)	
1		248 (23.8%)	144 (22.6%)	104 (25.7%)	
≥ 2		71 (6.8%)	48 (7.5%)	23 (5.7%)	
Synchronous mHSPC	1 208	1 033 (85.5%)	608 (95.1%)	425 (74.7%)	< 0.001
Visceral metastasis at mHSPC	1 215	148 (12.2%)	104 (16.3%)	44 (7.6%)	< 0.001
High‐volume mHSPC	1 107	749 (67.7%)	496 (77.6%)	253 (54.1%)	< 0.001
High‐risk mHSPC	1 117	797 (71.4%)	492 (77.4%)	305 (63.4%)	< 0.001
PSA at mHSPC (ng/ml)	1 073	139 (32, 580)	260 (74, 890)	43 (10, 240)	< 0.001
cT4 stage	931	205 (22.0%)	155 (24.9%)	50 (16.2%)	0.003
ISUP grade group	1 138				< 0.001
≤ GG3		173 (15.2%)	35 (5.7%)	138 (26.1%)	
GG4		285 (25.0%)	167 (27.4%)	118 (22.3%)	
GG5		680 (59.8%)	408 (66.9%)	272 (51.5%)	
First‐line treatment of mHSPC	1 215				< 0.001
ARPI		892 (73.4%)	485 (75.9%)	407 (70.7%)	
Docetaxel		153 (12.6%)	46 (7.2%)	107 (18.6%)	
ARPI+Docetaxel		170 (14.0%)	108 (16.9%)	62 (10.8%)	
Local therapy with RP/RT	1 097	195 (17.8%)	45 (7.0%)	150 (32.8%)	< 0.001
PSA nadir at mHSPC (ng/ml)	879	0.10 (0.01, 0.88)	0.07 (0.01, 0.72)	0.17 (0.03, 1.24)	< 0.001
Systemic therapy lines	1 213	2 (1, 2)	1 (1, 2)	2 (2, 3)	< 0.001
First‐line treatment of mCRPC	417				< 0.001
ARPI		227 (54.4%)	94 (57.7%)	133 (52.4%)	
Chemotherapy		132 (31.7%)	59 (36.2%)	73 (28.7%)	
PSMA‐RLT		34 (8.2%)	0 (0.0%)	34 (13.4%)	
Radium‐223		14 (3.4%)	9 (5.5%)	5 (2.0%)	
ARPI+PARP inhibitor		6 (1.4%)	0 (0.0%)	6 (2.4%)	
PARP inhibitor		4 (1.0%)	1 (0.6%)	3 (1.2%)	

^**a**^ Data are presented as median (IQR) or *n* (%). Percentages were calculated using the number of patients with available data for each variable.

^**b**^ Wilcoxon rank‐sum test, Pearson's Chi‐squared test.

### 3.2 Treatment Characteristics

The treatment variables for first‐line treatment differed significantly between patients in Japan and Germany (Table 1). As mHSPC treatment, ARPI‐based doublet therapy predominated in both cohorts (75.9% vs. 70.7%), while triple therapy was more frequent in Japan (16.9% vs. 10.8%) and docetaxel‐based doublet therapy was more frequent in Germany (7.2% vs. 18.6%, *p* < 0.001). Patients in Japan achieved higher PSA response under first‐line treatment (PSA nadir 0.07 vs. 0.17 ng/mL, *p* < 0.001). Moreover, patients in Japan received less local therapy for the primary prostate tumor (7.0% vs. 32.8%, *p* < 0.001).

After progression to mCRPC, Germany recorded greater use of later systemic lines. The observed median number of systemic therapies recorded was 1 (IQR 1–2) in Japan vs. 2 (IQR 2–3) in Germany. The subsequent treatment options in mCRPC also differed between patients in Japan and Germany (*p* < 0.001).

### 3.3 Time to mCRPC Analyses

**FIGURE 1:**
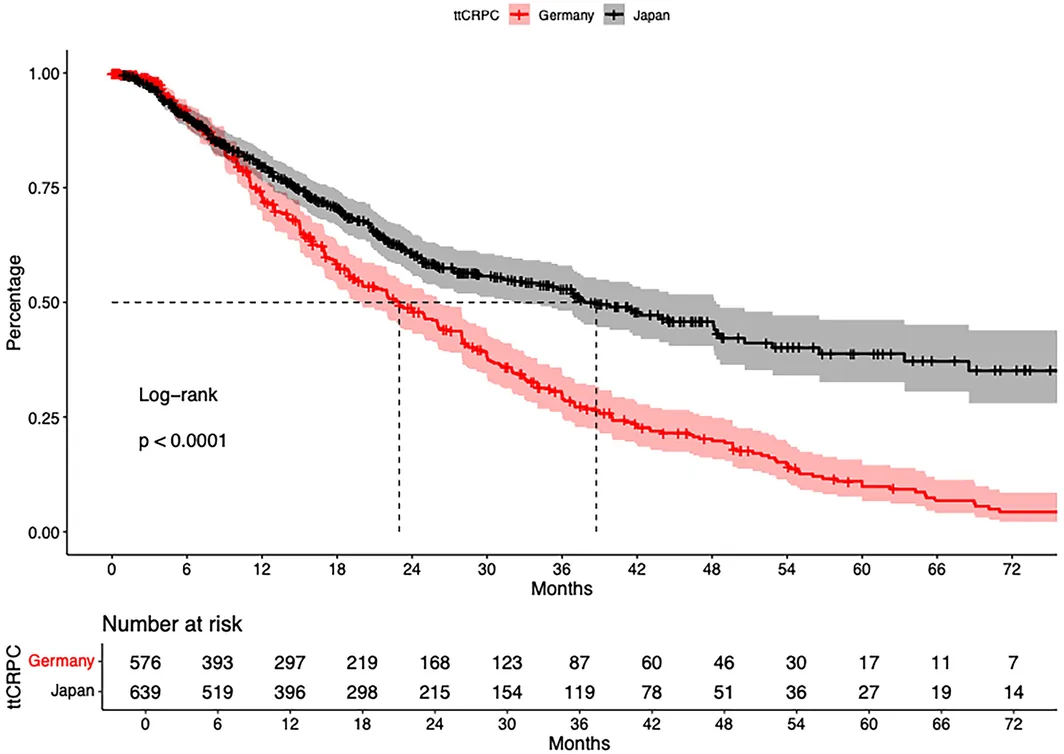
Kaplan–Meier curves depicting time to metastatic castration‐resistant prostate cancer (ttCRPC) of metastatic hormone‐sensitive prostate cancer patients in Japan vs. Germany.

In ttCRPC analyses, a significant difference in the comparison between patients in Japan vs. Germany was observed (*p* < 0.001), with a median ttCRPC of 39 (95% CI: 33–48) months for Japanese patients vs. 23 (95% CI: 20–26) months for German patients (Figure 1). The corresponding HR was 0.57 (95% CI: 0.48–0.67, *p* < 0.001, Table 2, Table S1). 12‐ and 24‐month mCRPC‐free survival rates were 79.6% vs. 72.9% and 60.6% vs. 48.4% in Japanese vs. German mHSPC patients. For the ttCRPC models, evidence of non‐proportionality was observed for the country variable, consistent with the Kaplan–Meier curves that remained largely overlapping during the first year before gradually diverging thereafter. Accordingly, the reported hazard ratios for ttCRPC should be interpreted as average effects over the follow‐up period.

**TABLE 2:** Univariable and multivariable adjusted Cox regression models predicting time to castration resistant prostate cancer and overall survival according to Japan vs. Germany.

Japan (Ref. Germany)	Univariable			Multivariable^a^		
	HR	CI	*p*	HR	CI	*p*
Time to castration resistant prostate cancer	0.57	0.48–0.67	< 0.001	0.52	0.40–0.67	< 0.001
Overall survival^b^	0.69	0.55–0.85	< 0.001	0.54	0.39–0.77	< 0.001

^a^ Adjusted for year of mHSPC treatment initiation, age at mHSPC diagnosis, ECOG, first‐line systemic treatment, PSA at mHSPC, local treatment, disease volume, and synchronous vs. metachronous metastatic disease.

^b^ Additionally adjusted for the number of systemic therapy lines for prostate cancer.

After further multivariable adjustment in Cox regression models, Japan was identified as an independent predictor for longer ttCRPC with an HR of 0.52 (CI: 0.40–0.67, *p* < 0.001, Table 2) relative to German mHSPC patients. These findings were consistent in sensitivity analyses using propensity score matched cohorts (Table S2 and Table S3, Figure S1 and Figure S2).

### 3.4 Overall Survival Analyses

**FIGURE 2:**
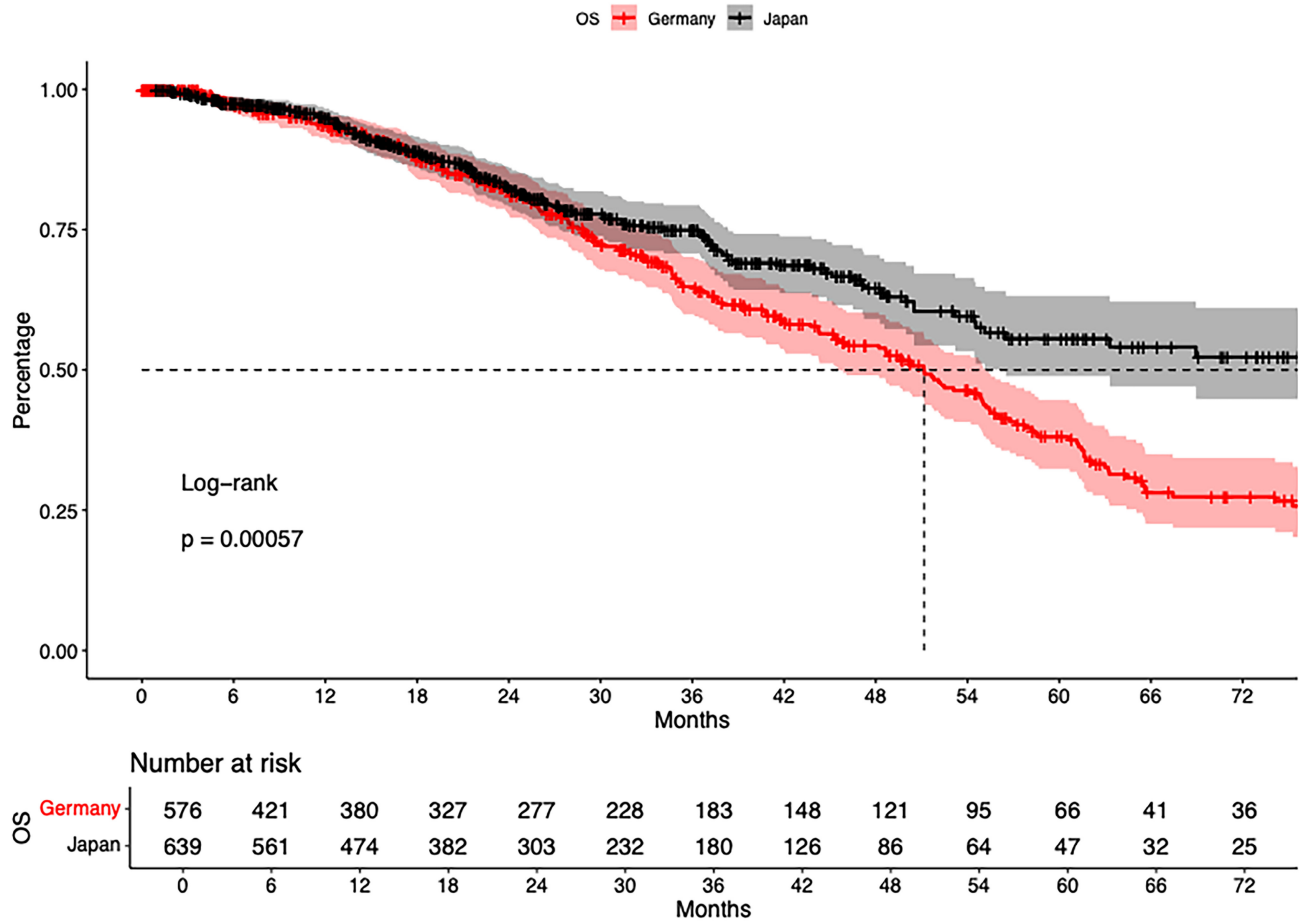
Kaplan–Meier curves depicting overall survival (OS) of metastatic hormone‐sensitive prostate cancer patients in Japan vs. Germany.

In OS analyses, significant differences in the comparison between patients in Japan vs. Germany were observed (*p* < 0.001), with median OS of 80 (95% CI: 57–not reached) months for Japanese patients vs. 51 (95% CI: 46–55) months for German patients (Figure 2). The corresponding HR was 0.69 (CI: 0.55–0.85, *p* < 0.001, Table 2, Table S1). 60‐month overall survival rates were 55.6% in Japanese patients vs. 38.0% in German mHSPC patients. The proportional hazards assumption was fulfilled for the overall survival models.

After further multivariable adjustment in Cox regression models, Japan was identified as an independent predictor for better OS with an HR of 0.54 (CI: 0.39–0.77, *p* < 0.001, Table 2) relative to German mHSPC patients. These findings were consistent in sensitivity analyses using propensity score matched cohorts (Table S2 and Table S3, Figure S1 and Figure S3).

## 4 Discussion

In this real‐world cross‐country analysis, we compared two large, well‐characterized cohorts of patients with mHSPC from Germany and Japan, derived from the FRAMCAP and JIKEI‐YAYOI databases. The FRAMCAP database has previously been used to investigate treatment patterns and outcomes in metastatic prostate cancer within a European tertiary care setting [20, 21]. In addition, the JIKEI‐YAYOI database has provided insights into treatment strategies and outcomes in multi‐institutional studies [22, 23]. Comparable findings have been reported in other real‐world studies from both Western and Asian cohorts [1, 24, 25, 26, 27]. However, direct comparisons between Western and Asian real‐world mHSPC populations remain scarce, as available evidence is mostly derived from post hoc subgroup analyses of randomized trials [11, 12, 13, 19].

First, we observed substantial differences in baseline characteristics between both cohorts. Patients in Japan presented with more advanced disease, including higher PSA at mHSPC, a higher proportion of synchronous metastatic disease, increased rates of high‐volume disease, and more frequent visceral metastases. These findings are consistent with previous reports from Japanese real‐world cohorts, which have described a higher burden of metastatic disease at diagnosis compared to Western populations. For example, Yasuda et al. reported 77% and 73% of patients presenting with high‐volume and high‐risk disease, respectively, similar to our findings in the Japanese cohort (78% and 77%) [24]. Conversely, German and other European real‐world studies have reported a lower burden of metastatic disease at diagnosis. Several structural differences between both healthcare systems may contribute to these observations, including differences in PSA screening uptake, opportunistic vs. organized early detection strategies, referral pathways to specialized centers, access to diagnostic imaging, and reimbursement policies. Such factors may influence the stage at diagnosis and contribute to stage migration, thereby affecting the apparent baseline disease burden observed in real‐world cohorts. These healthcare‐related differences should therefore be considered when interpreting cross‐country comparisons of oncologic outcomes.

Second, treatment patterns differed between the Japanese and German cohorts. While ARPI‐based doublet therapy represented the predominant first‐line treatment in both countries, the use of triple therapy was higher in Japan, whereas docetaxel‐based treatment without ARPI was more frequently used in Germany. In addition, local treatment of the primary tumor was substantially more common in the German cohort (33%) compared to the Japanese cohort (7.0%), which is consistent with findings from subgroup analyses of the ARCHES and TITAN trials, where rates of prior local therapy were also higher in Western (33% and 19%) compared to Asian patients (4.3% and 5.4%) [11]. Moreover, these observations are consistent with previous real‐world analyses demonstrating marked cross‐country differences in treatment patterns in mHSPC, largely driven by variations in drug approval status, guideline endorsement, reimbursement policies, and treatment availability [28, 29].

Third, despite less favorable baseline characteristics, patients in the Japanese cohort exhibited significantly prolonged ttCRPC and OS compared with their German counterparts. Importantly, these associations persisted after comprehensive multivariable adjustment as well as propensity score matching, indicating that the measured clinical and treatment‐related variables do not fully explain the observed differences between both cohorts. Consequently, the present analyses cannot identify the underlying mechanisms responsible for these outcome differences but rather suggest that additional unmeasured biological, behavioral, or healthcare‐related factors may contribute. The results of the current study are in line with post hoc analyses of randomized trials, which reported favorable outcomes in Asian subgroups treated with ARPI‐based doublet therapies. For example, in LATITUDE, 36‐month progression‐free survival and OS rates in the abiraterone plus ADT arm were approximately 75% and 91% in Japanese patients compared to 60% and 66% in the overall population [12, 13]. In TITAN, 36‐month mCRPC‐free survival and OS rates in the apalutamide plus ADT arm were approximately 68% and 76% in Asian patients compared to 64% and 72% in the overall population [14]. In ARCHES, 12‐month mCRPC‐free survival rates in the enzalutamide plus ADT arm were approximately 90% in Japanese patients compared to 84% in the overall population [11]. These findings support the hypothesis that differences in tumor biology, ethnicity, pharmacogenomics, diet, physical activity, social environment, or treatment adherence may contribute to outcome variability between populations, as previously suggested by studies demonstrating inter‐ethnic differences in genetic background and treatment response [30]. Also, differences in subsequent treatment patterns after progression to mCRPC cannot explain the longer OS in the Japanese cohort, as German patients in the current study received a higher number of subsequent systemic therapy lines.

Finally, several limitations of this study should be acknowledged. First, the retrospective design introduces the risk of selection bias and residual confounding despite comprehensive multivariable adjustment and propensity score matching. Second, although harmonized inclusion criteria were applied, this cross‐country comparison inevitably reflects differences between the German and Japanese healthcare systems. Variations in PSA screening practices, referral pathways, drug reimbursement policies, access to advanced imaging modalities such as PSMA PET/CT, and staging strategies may have influenced patient selection, baseline disease characteristics, treatment allocation, and clinical outcomes. Consequently, residual confounding related to structural healthcare differences cannot be completely excluded. Third, unmeasured confounders, including comorbidity burden, frailty, socioeconomic and lifestyle factors, treatment adherence, and molecular tumor characteristics, were not consistently available in both databases and therefore could not be incorporated into the analyses. Finally, because of the retrospective design and differences in data collection between both registries, some clinically relevant variables could not be assessed in greater detail, including the timing and type of local treatment as well as detailed metastatic substage. Accordingly, the observed differences between both cohorts should be interpreted as associations rather than evidence of causal relationships.

Taken together, this study provides the first direct real‐world comparison between Western and Asian mHSPC populations using large contemporary datasets. Despite presenting with more adverse baseline characteristics, patients in Japan demonstrated significantly prolonged ttCRPC and improved OS compared with patients in Germany. These differences may reflect not only potential biological variations but also differences in screening, staging, and treatment strategies. These findings highlight the need for further research into population‐specific factors, including biological and healthcare system‐related determinants, to optimize treatment strategies for patients with mHSPC worldwide. They are also of great importance when planning large international Phase III trials.

## Author Contributions

**Mike Wenzel:** conceptualization, writing – original draft, data curation, methodology, investigation. **Philipp Mandel:** conceptualization, investigation, methodology, writing – original draft, data curation. **Keiichiro Miyajima:** conceptualization, investigation, methodology, data curation, writing – original draft. **Maximilian Filzmayer:** formal analysis, writing – review and editing, data curation. **Takafumi Yanagisawa:** validation, supervision, writing – review and editing. **Benedikt Lauer:** data curation, formal analysis. **Shin Koike:** validation, supervision, writing – review and editing. **Axel S. Merseburger:** supervision, writing – review and editing, validation. **Takahiro Kimura:** validation, supervision, writing – review and editing. **Carolin Siech:** methodology, investigation, conceptualization. **Fumihiko Urabe:** conceptualization, investigation, data curation, writing – original draft, methodology. **Yoichiro Tohi:** validation, supervision, writing – review and editing. **Kojiro Tashiro:** validation, supervision, writing – review and editing. **Felix K.‐H. Chun:** conceptualization, supervision, validation, writing – review and editing.

## Ethics Statement

The current study was in accordance with the Declaration of Helsinki. Review by the local ethics boards in Germany (reference no. SUG‐5‐2024) and Japan (reference no. 37–562‐13 219) was approved. Given the retrospective design and anonymized data, the requirement for individual patient consent was waived.

## Consent

The authors have nothing to report.

## Conflicts of Interest

M.W. receives speaker honoraria or is consultant for Accord, Astellas, Johnson & Johnson, Pfizer, MSD, Astra Zeneca, Ipsen, Novartis and Bayer. M.F. receives speaker honoraria or is consultant for Johnson & Johnson and Bayer. AM receives honoraria or is consultant for Ambu, Amgen, Apogepha, AstraZeneca, Astellas, Bayer, Bristol‐Myers Squibb, Eisai, EUSAPharma, Farco, Ferring Ipsen, Hexal, Sandoz, MedUpdate, MSD, Merck Serono, Novartis, Janssen, Pfizer, Takeda, Novartis, Recordati and Roche. FC receives speaker honoraria or is consultant for Astellas, AstraZeneca, Bayer, Johnson & Johnson, Boston Scientific, Novartis, Molecular Health, Olympus and Pfizer. T.K. receives speaker honoraria or is consultant for Astellas, AstraZeneca, Bayer, Janssen, Sanofi, and Takeda. F.U. receives speaker honoraria or is consultant for Astellas, AstraZeneca, Bayer, Johnson & Johnson, and Takeda. P.M. receives speaker honoraria or is consultant for AMGEN, Astellas, AstraZeneca, Bayer, IPSEN, Johnson & Johnson, MSD, Novartis, Orion and Pfizer.

## Supporting information


**Table S1:** Univariable and multivariable adjusted Cox regression models predicting time to castration resistant prostate cancer and overall survival according to Japan vs. Germany, presenting all covariates.
**Table S2:** Descriptive characteristics of propensity score‐matched (Japan vs. Germany) metastatic hormone‐sensitive prostate cancer patients.
**Figure S1:** Love plot illustrating covariate balance before (unadjusted) and after (adjusted) propensity score matching.
**Figure S2:** Kaplan–Meier curves depicting time to metastatic castration‐resistant prostate cancer (ttCRPC) of metastatic hormone‐sensitive prostate cancer patients in Japan vs. Germany in the 1:1 propensity score‐matched cohort.
**Figure S3:** Kaplan–Meier curves depicting overall survival (OS) of metastatic hormone‐sensitive prostate cancer patients in Japan vs. Germany in the 1:1 propensity score‐matched cohort.
**Table S3:** Cox regression models for time to castration‐resistant prostate cancer and overall survival in the 1:1 propensity score‐matched cohort (Japan vs. Germany), including unadjusted and covariate‐adjusted (doubly robust) analyses with robust standard errors accounting for matched subclasses.

## Data Availability

The data that support the findings of this study are available from the corresponding author upon reasonable request.
