# Supplementary material for: Cancer‐Control Outcomes of Japanese Vs. German Metastatic Hormone‐Sensitive Prostate Cancer Patients: A Real‐World Cross‐Country Comparison of Patients Undergoing Combination Therapies
Source: Int J Urol. 2026 Sep 9;33(9):e70633. doi: 10.1111/iju.70633 (PMC13558785; doi:10.1111/iju.70633)
Supplement: Supplementary file 1 [file IJU-33-0-s001.docx]

**Supplementary Table 1.** Univariable und multivariable adjusted Cox regression models predicting time to castration resistant prostate cancer and overall survival according to Japan versus Germany, presenting all covariates.

|  | Time to castration resistant prostate cancer | | | Overall survival | | |
| --- | --- | --- | --- | --- | --- | --- |
|  | **HR** | **CI** | **p-value** | **HR** | **CI** | **p-value** |
| Unadjusted |  | | |  | | |
| Japan (Ref. Germany) | 0.57 | 0.48–0.67 | <0.001 | 0.69 | 0.55–0.85 | <0.001 |
| Covariate-adjusted |  | | |  | | |
| Japan (Ref. Germany) | 0.52 | 0.40–0.67 | <0.001 | 0.54 | 0.39–0.77 | <0.001 |
| Year of mHSPC treatment initiation | 0.99 | 0.93–1.06 | 0.8 | 1.03 | 0.94–1.12 | 0.6 |
| Age at mHSPC diagnosis (per year) | 1.02 | 1.01–1.04 | <0.001 | 1.03 | 1.02–1.05 | <0.001 |
| ECOG 1 (Ref. ECOG 0) | 1.14 | 0.89–1.46 | 0.3 | 1.36 | 0.99–1.86 | 0.1 |
| ECOG ≥2 (Ref. ECOG 0) | 1.30 | 0.88–1.91 | 0.2 | 2.24 | 1.44–3.49 | <0.001 |
| First-line Docetaxel (Ref. ARPI) | 1.60 | 1.18–2.17 | 0.002 | 0.94 | 0.65–1.36 | 0.7 |
| First-line ARPI+Docetaxel (Ref. ARPI) | 0.67 | 0.41–1.09 | 0.1 | 0.82 | 0.41–1.64 | 0.6 |
| PSA at diagnosis (per ng/ml) | 1.00 | 1.00–1.00 | 0.4 | 1.00 | 1.00–1.00 | 0.8 |
| Local treatment (yes vs. no) | 0.90 | 0.60–1.33 | 0.6 | 1.17 | 0.72–1.89 | 0.5 |
| High-volume mHSPC (Ref. Low-volume) | 1.48 | 1.11–1.98 | 0.008 | 1.67 | 1.14–2.43 | 0.009 |
| Synchronous mHSPC (Ref. metachronous) | 1.35 | 0.83–2.19 | 0.2 | 1.29 | 0.73–2.28 | 0.4 |
| Number of systemic therapy lines | - | - | - | 1.13 | 1.02–1.25 | 0.017 |

**Supplementary Table 2.** Descriptive characteristics of propensity score-matched (Japan vs. Germany) metastatic hormone-sensitive prostate cancer patients

| Characteristic | N | Japan  N = 257  (50.0%)^a^ | Germany  N = 257  (50.0%)^a^ |
| --- | --- | --- | --- |
| Year of mHSPC treatment initiation | 514 | 2022 (2020, 2023) | 2022 (2020, 2024) |
| Age at mHPSC diagnosis (years) | 514 | 71 (65, 76) | 70 (64, 76) |
| ECOG status | 514 |  |  |
| 0 |  | 176 (68.5%) | 181 (70.4%) |
| 1 |  | 66 (25.7%) | 62 (24.1%) |
| ≥2 |  | 15 (5.8%) | 14 (5.4%) |
| Synchronous mHSPC | 514 | 230 (89.5%) | 223 (86.8%) |
| Visceral metastasis at mHSPC | 514 | 33 (12.8%) | 22 (8.6%) |
| High-volume mHSPC | 514 | 162 (63.0%) | 158 (61.5%) |
| High-risk mHSPC | 514 | 173 (67.3%) | 173 (67.8%) |
| PSA at mHSPC (ng/ml) | 514 | 159 (48, 497) | 81 (17, 360) |
| cT4 stage | 422 | 62 (24.8%) | 33 (19.2%) |
| ISUP grade group | 487 |  |  |
| ≤GG3 |  | 13 (5.2%) | 58 (24.4%) |
| GG4 |  | 60 (24.1%) | 41 (17.2%) |
| GG5 |  | 176 (70.7%) | 139 (58.4%) |
| First line treatment of mHSPC | 514 |  |  |
| ARPI |  | 181 (70.4%) | 179 (69.6%) |
| Docetaxel |  | 37 (14.4%) | 36 (14.0%) |
| ARPI+Docetaxel |  | 39 (15.2%) | 42 (16.3%) |
| Local therapy with RP/RT | 480 | 37 (14.4%) | 58 (26.0%) |
| PSA nadir at mHSPC (ng/ml) | 408 | 0.05 (0.01, 0.52) | 0.27 (0.03, 1.51) |
| Systemic therapy lines | 514 | 1 (1, 2) | 2 (2, 2) |
| First line treatment of mCRPC | 152 |  |  |
| ARPI |  | 38 (55.1%) | 41 (49.4%) |
| Chemotherapy |  | 25 (36.2%) | 27 (32.5%) |
| PSMA-RLT |  | 0 (0.0%) | 10 (12.0%) |
| Radium-223 |  | 5 (7.2%) | 2 (2.4%) |
| ARPI+PARP inhibitor |  | 0 (0.0%) | 2 (2.4%) |
| PARP inhibitor |  | 1 (1.4%) | 1 (1.2%) |

**^a^** Data are presented as median (IQR) or n (%).

**Supplementary Figure 1.** Love plot illustrating covariate balance before (unadjusted) and after (adjusted) propensity score matching

**Supplementary Figure 2.** Kaplan Meier curves depicting time to metastatic castration resistant prostate cancer (ttCRPC) of metastatic hormone-sensitive prostate cancer patients in Japan vs. Germany in the 1:1 propensity score-matched cohort.

**Supplementary Figure 3.** Kaplan Meier curves depicting overall survival (OS) of metastatic hormone-sensitive prostate cancer patients in Japan vs. Germany in the 1:1 propensity score-matched cohort.

**Supplementary Table 3.** Cox regression models for time to castration-resistant prostate cancer and overall survival in the 1:1 propensity score-matched cohort (Japan vs. Germany), including unadjusted and covariate-adjusted (doubly robust) analyses with robust standard errors accounting for matched subclasses.

|  | Time to castration resistant prostate cancer | | | Overall survival | | |
| --- | --- | --- | --- | --- | --- | --- |
|  | **HR** | **CI** | **p-value** | **HR** | **CI** | **p-value** |
| Unadjusted |  | | |  | | |
| Japan (Ref. Germany) | 0.58 | 0.45–0.77 | <0.001 | 0.47 | 0.33–0.66 | <0.001 |
| Covariate-adjusted |  | | |  | | |
| Japan (Ref. Germany) | 0.55 | 0.41–0.73 | <0.001 | 0.43 | 0.28–0.65 | <0.001 |
| Year of mHSPC treatment initiation | 0.97 | 0.90–1.06 | 0.5 | 1.07 | 0.93–1.22 | 0.4 |
| Age at mHSPC diagnosis (per year) | 1.02 | 1.00–1.04 | 0.037 | 1.01 | 0.99–1.03 | 0.5 |
| ECOG 1 (Ref. ECOG 0) | 1.08 | 0.80–1.47 | 0.6 | 1.56 | 1.03–2.37 | 0.038 |
| ECOG ≥2 (Ref. ECOG 0) | 1.40 | 0.82–2.39 | 0.2 | 2.87 | 1.48–5.59 | 0.002 |
| First-line Docetaxel (Ref. ARPI) | 1.48 | 1.02–2.15 | 0.040 | 1.04 | 0.67–1.61 | 0.9 |
| First-line ARPI+Docetaxel (Ref. ARPI) | 0.69 | 0.39–1.21 | 0.2 | 0.84 | 0.37–1.91 | 0.7 |
| PSA at diagnosis (per ng/ml) | 1.00 | 1.00–1.00 | 0.011 | 1.00 | 1.00–1.00 | 0.7 |
| Local treatment (yes vs. no) | 0.82 | 0.54–1.26 | 0.4 | 0.88 | 0.51–1.52 | 0.6 |
| High-volume mHSPC (Ref. Low-volume) | 1.64 | 1.15–2.33 | 0.006 | 1.51 | 0.93–2.46 | 0.1 |
| Synchronous mHSPC (Ref. metachronous) | 1.39 | 0.86–2.24 | 0.2 | 1.45 | 0.82–2.59 | 0.2 |
| Number of systemic therapy lines | - | - | - | 1.04 | 0.91–1.20 | 0.6 |
